# Supplementary material for: Optogenetic control of YAP reveals a dynamic communication code for stem cell fate and proliferation
Source: Nat Commun. 2023 Oct 30;14:6929. doi: 10.1038/s41467-023-42643-2 (PMC10616176; doi:10.1038/s41467-023-42643-2)
Supplement: Supplementary file 1 — Supplementary Information [file 41467_2023_42643_MOESM1_ESM.pdf]

## Supplementary information

# Optogenetic control of YAP reveals a dynamic communication code for stem cell fate and proliferation

Kirstin Meyer[1,2], Nicholas C. Lammers[3,4], Lukasz J. Bugaj[5], Hernan G. Garcia[3,6,7,8,9], Orion D. Weiner[1,2*]

[1]Cardiovascular Research Institute, University of California, San Francisco, San Francisco, CA 94158, USA
[2]Department of Biochemistry and Biophysics, University of California, San Francisco, San Francisco, CA 94158, USA.
[3]Biophysics Graduate Group, University of California at Berkeley, Berkeley, CA 94720, USA
[4]Department of Genome Sciences, University of Washington, Seattle, WA 98195, USA
[5]Department of Bioengineering, University of Pennsylvania, Philadelphia, PA 19104, USA
[6]Department of Physics, University of California at Berkeley, Berkeley, CA 94720, USA
[7]Department of Molecular and Cell Biology, University of California Berkeley, Berkeley, CA 94720, USA
[8]Institute for Quantitative Biosciences-QB3, University of California at Berkeley, Berkeley, CA 94720, USA
[9]Chan Zuckerberg Biohub, San Francisco, CA 94158, USA

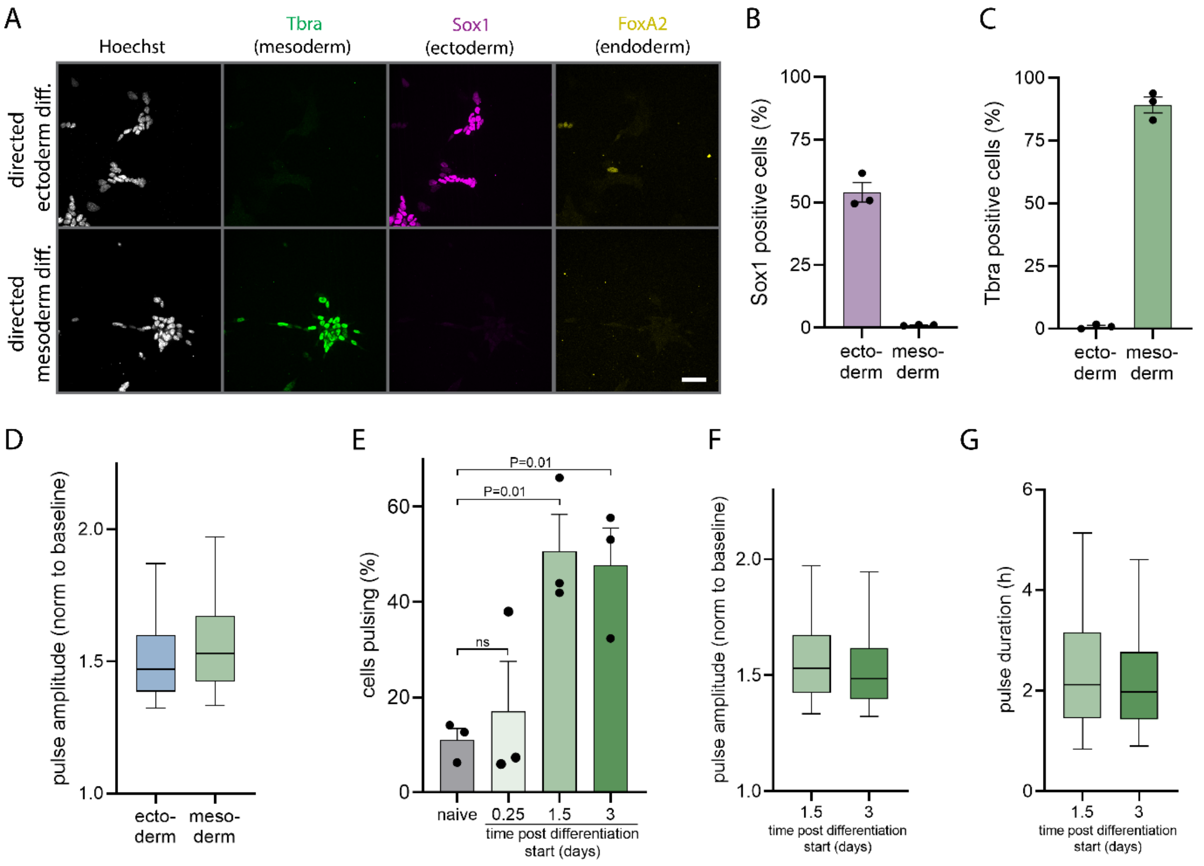


**Supplementary Figure 1 Characterization of endogenous YAP dynamics during mESC differentiation**

**A)** IF staining for differentiation markers Sox1 (ectoderm), Tbra (mesoderm) and FoxA2 (endoderm) at 5d post directed differentiation into the ecto- and mesoderm lineage. Images are representative from N=3 independent experiments. Scale bar: 50 µm **B, C**) Quantification of Sox1 and Tbra positive cells upon directed differentiation into the ectoderm (B) and mesoderm (C) lineages. Markers were quantified from IF images as shown in (A). Shown are mean +/- SEM, N=3 independent experiments. **D**) Quantification of pulse amplitude in mESCs directed along the ectoderm or mesoderm lineages at 1.5d post differentiation start. Pulses were classified by the peak detection strategy shown in Supplementary Fig. 2. Shown are the Box and Whiskers with median and 5-95 Percentile, data pooled from three independent experiments with a total of 621 (mesoderm) and 410 (ectoderm) cells. (D). **E-G**) Quantification of endogenous YAP dynamics over the time course of early mesoderm differentiation. Shown are the percentage of cells exhibiting YAP pulses (E) and the amplitude (F) and duration (G) of those pulses at 1.5d and 3d post differentiation. Data for the naive condition and 1.5d post differentiation start in panels E-G are replicated from Fig. 1E, F and panel D. Shown are mean +/- SEM from N=3 independent experiments. (E) and the Box and Whiskers with median and 5-95 Percentile. In F and G, data was pooled from N=3 independent experiments, p values from two-sided unpaired Student's t test (E).

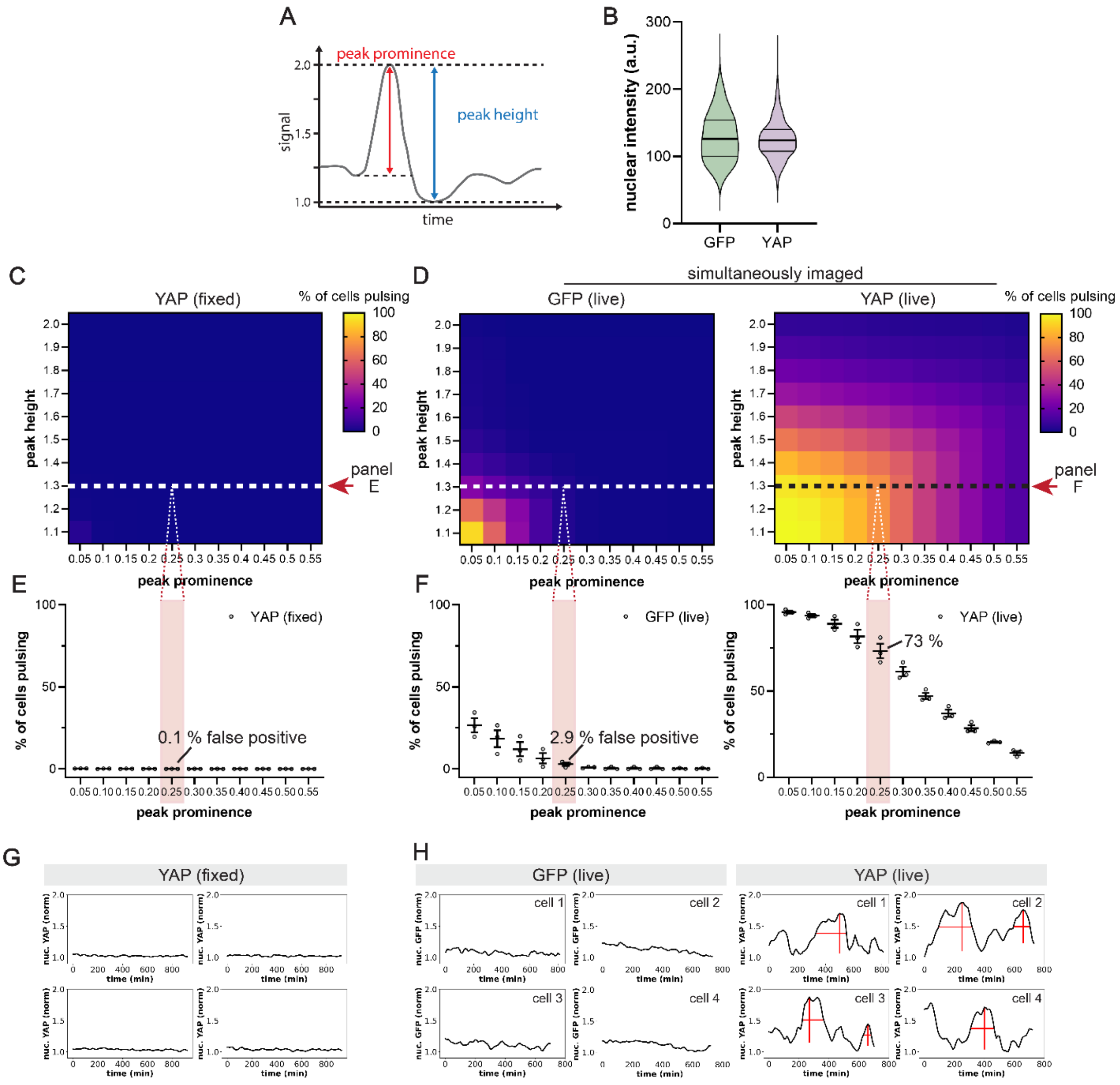


## Supplementary Figure 2 Performance verification of the peak detection algorithm

**A**) The peak detection algorithm identifies local maxima (peak prominence, red arrow) by comparison of neighboring values and further filters identified peaks by the absolute peak height (blue arrow). **B**) Quantitative comparison of the absolute GFP intensity to the endogenous YAP signal demonstrates comparable signal-to-noise for both reporters. Data pooled from N=3 independent experiments with a total of 546 data points each. **C-H**) Verification of our peak detection approach by comparison of endogenous YAP pulses to fixed cells expressing endogenous SNAP-YAP (panel C, E, G) and simultaneous live-imaging of a non-dynamic fluorophore (GFP) and endogenous YAP (panel D, F, H) at comparable signal-to-noise (see B). Heatmaps (C, D) show the percentage of cells pulsing as a function of peak prominence and peak height. Shown are mean, N=3 independent experiments. Panels E and F refer to the white dashed line in panels C, D and show the effect of peak prominence for pulse detection at fixed peak height = 1.3. Our parameter choice (prominence = 0.25, peak height = 1.3, red shading in panels E,F) yields a false positive rate of 4% (GFP control: 2.9% of cells pulsing; YAP: 73% of cells pulsing). Example traces of all conditions (fixed YAP, GFP control and endogenous YAP) are shown in G, H. The GFP and YAP traces were acquired simultaneously in the same cells (see cell ID label). Peak height and width are indicated by horizontal and vertical red lines. Panel E,F show mean +/- SEM, N=3 independent experiments.

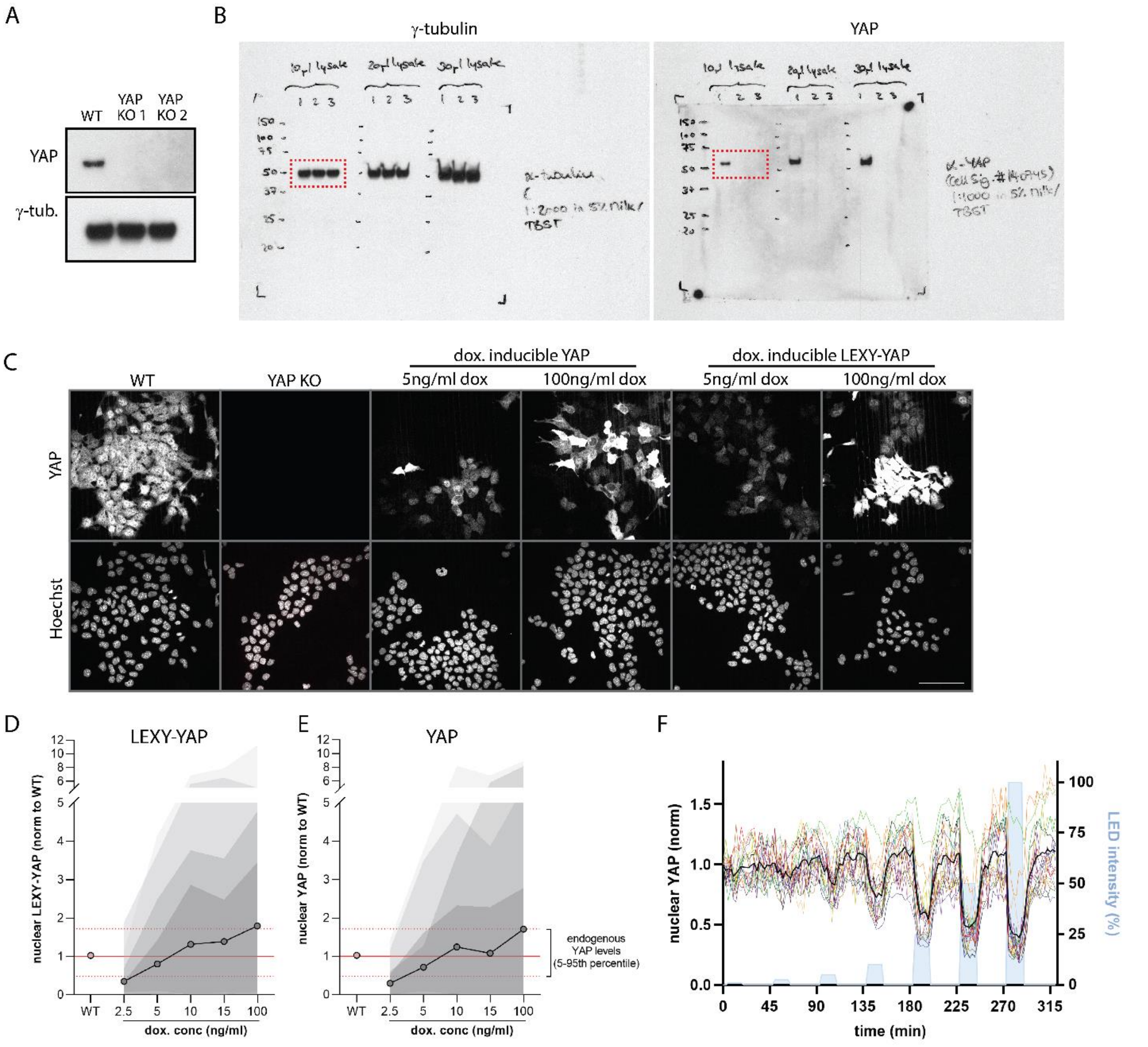


## Supplementary Figure 3 Characterization of the inducible and light-gated LEXY-YAP tool

**A**) Western blot detection of YAP protein levels in WT and YAP knockout (KO) mESCs shows depletion of YAP protein in two different clonal KO lines generated with different CRISPR guides. γ-tubulin serves as loading control. **B**) Uncropped Western blots. Red dashed rectangles indicate parts shown in (A). **C**) Representative IF staining (from N=4 independent experiments) of YAP in WT, YAP KO and doxycycline induced YAP or LEXY-YAP mESCs with indicated concentrations shows titratability of the YAP and LEXY-YAP tools. WT cells serve as reference for endogenous YAP levels. The YAP and LEXY-YAP constructs were expressed in the YAP KO background. Scale bar: 100 µm. **D, E**) Doxycycline induction of LEXY-YAP (C) and YAP (D) in YAP KO mESCs provides access to a wide expression range bracketing the endogenous YAP levels of WT mECS. Induction was performed with indicated doxycycline concentrations and quantified from IF images as representatively shown in (C) at 2d post spontaneous differentiation. The mean (solid red line) + 5-95 percentiles (dashed red lines) of endogenous YAP levels are shown. LEXY-YAP and YAP levels are shown as mean (solid black line) and the 5-95th percentile of the induction range from N=4 independent experiments (gray shading) are shown as overlay. **F**) Quantification of nuclear LEXY-YAP levels upon consecutive illumination cycles with different LED intensities demonstrates titratable nuclear YAP export. Data was quantified from microscopy time series of one experiment. Illumination phases are indicated by blue shading. Colored traces represent single cells, and the black solid line represents the population mean, N=1 experiment.

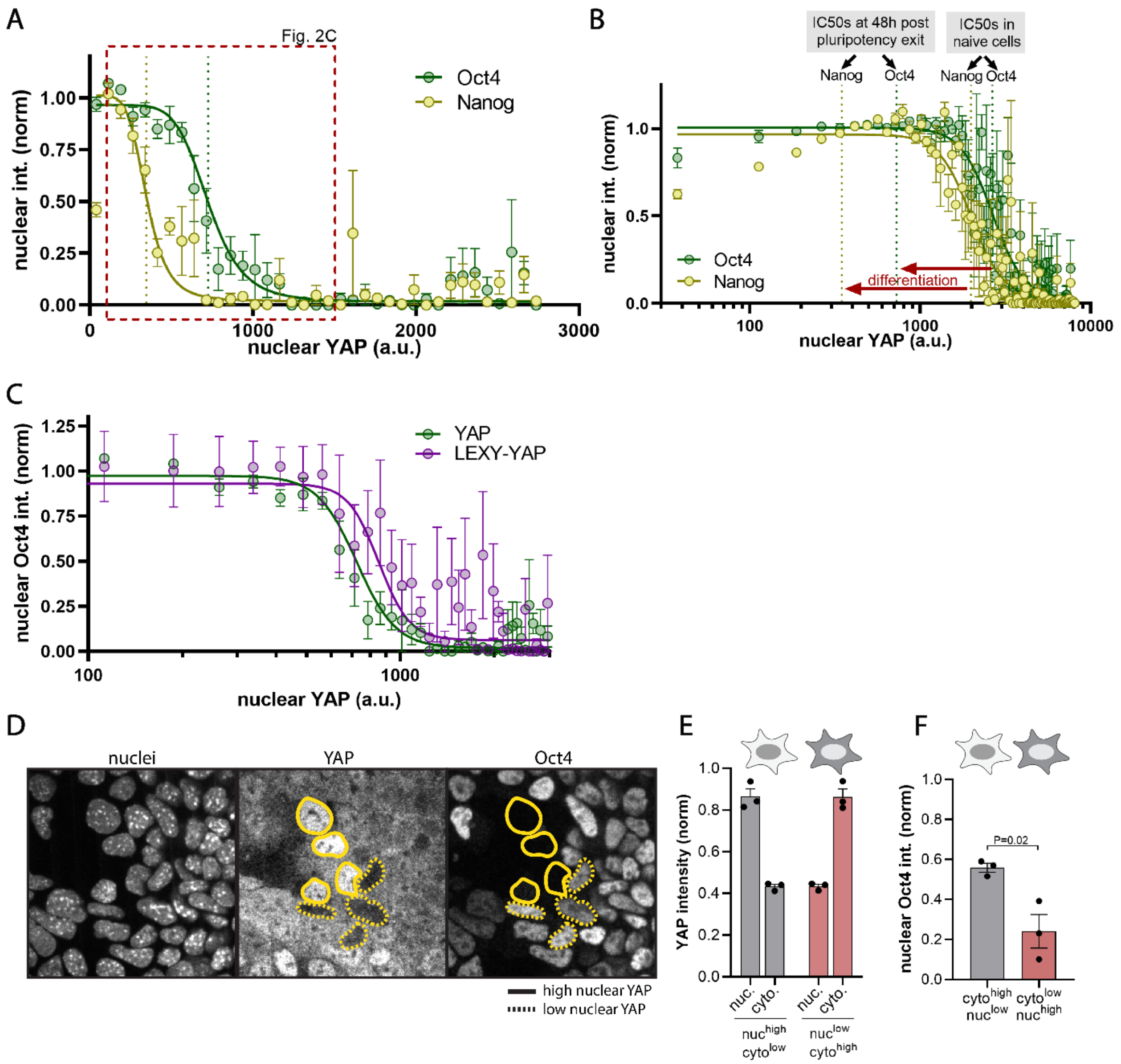


## Supplementary Figure 4 Quantification of YAP-dependent Oct4 and Nanog repression

**A**) Full sigmoidal curve fit of nuclear Nanog and Oct4 levels at 48h post undirected differentiation. Zoomed in part shown in Fig. 2C is outlined by red dashed rectangle. See legend of Fig. 2C for details. **B**) Sigmoidal curve fit of nuclear Nanog and Oct4 levels as a function of nuclear YAP concentrations under naïve conditions (2i+LIF) reveals shifted repression regimes (red arrows) as compared to differentiation conditions (48h post pluripotency exit). IC50s in naïve cells and 48h post pluripotency exit for Nanog and Oct4 are indicated. The IC50s at 48h post pluripotency exit represent the data shown in Fig. 2C. Shown are mean +/- SEM, N=3 independent experiments. **C**) Comparison of the sigmoidal curve fits of nuclear Oct4 levels as a function of nuclear YAP (green) or LEXY-YAP (magenta) concentrations show comparable repressive potency of both constructs. Shown are mean +/- SEM, N=4 independent experiments. **D-F**) Role of cytoplasmic vs. nuclear YAP for Oct4 repression by comparison of Oct4 levels in differentiating mESCs with high nuclear and low cytoplasmic YAP (panel D, solid yellow line; panel E,F grey bars), or low nuclear and high cytoplasmic YAP (panel D, dashed yellow line; panel E,F red bars). The YAP and Oct4 signal were quantified from IF stainings as representatively shown in (D), and cells were selected for comparable YAP levels in the opposing compartment (E). Representative images in (D) were chosen from N=3 independent experiments. Oct4 levels are significantly lower in presence of high nuclear YAP than comparable cytoplasmic levels (F). Shown are mean +/- SEM, N=3 independent experiments (E,F). p values from two-sided unpaired Student's t test (F).

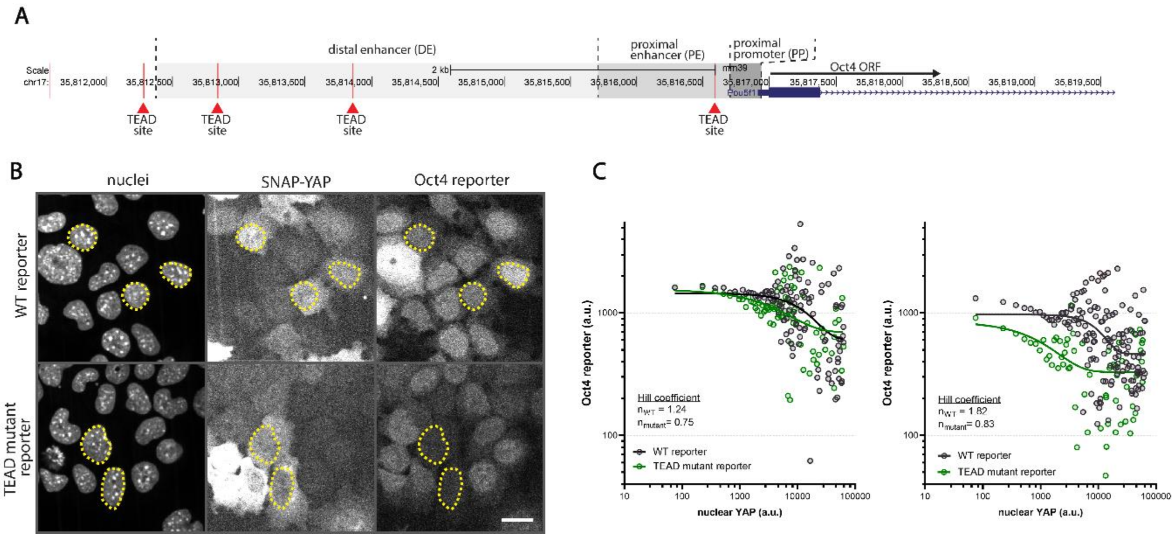


## Supplementary Figure 5 Oct4 locus map and TEAD reporter imaging

**A**) UCSC genome browser map[1] of the Oct4 regulatory region. The locus is controlled by a proximal promoter as well as a distal and proximal enhancer, their respective locations are indicated as previously defined[2]. TEAD sites predicted by the JASPAR database[3] and identified by a previous report[4] are indicated in red. **B**) Images of the Oct4-GFP reporter harboring the WT locus sequence (WT reporter) or in absence of the four TEAD binding sites (TEAD mutant reporter) shows higher sensitivity of the Oct4 reporter to low YAP levels. Nuclei outlined with yellow dashed lines show cells with comparable YAP levels but different GFP reporter repression. See quantification in panel C and Fig. 2D. Scale bar, 20 μm (B). Representative images were chosen from N=3 independent experiments. **C**) Quantification and sigmoidal fit of the WT and TEAD mutant reporter as a function of nuclear YAP levels. Left and right show two independent experiments in addition to the one shown in Fig. 2D. Hill coefficients from fits are shown.

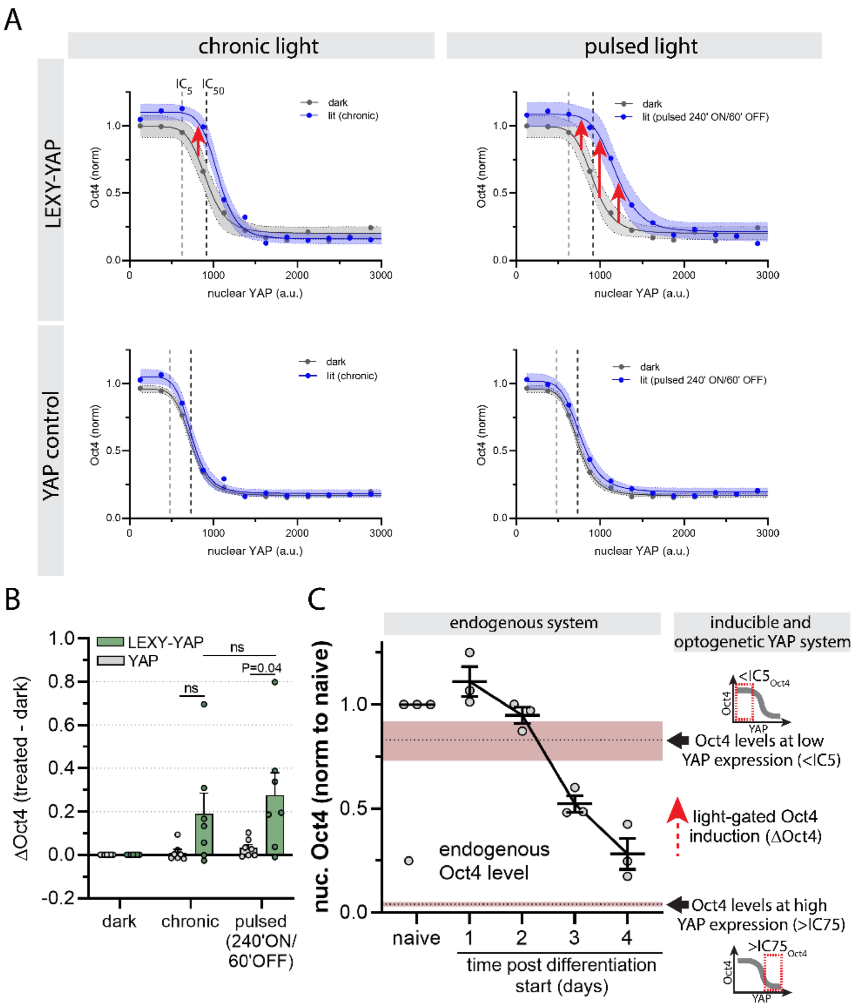


**Supplementary Figure 6 Quantification of Oct4 induction upon light gated control of YAP export**

**A**) Sigmoidal curve representation of the light-gated Oct4 induction shown in Fig. 3C. Shown are Oct4 protein levels as a function of nuclear LEXY-YAP/YAP levels upon chronic (top left) and pulsed (240min ON/60min OFF; top, right) illumination in LEXY-YAP (top) and YAP control cells (bottom). The YAP control cells express the same SNAP-YAP construct as the LEXY-YAP cells but lack the light-sensitive LEXY-tag. All light conditions (blue) are shown in reference to a dark control (grey). Shown are mean from N=7 independent experiments and the sigmoidal curve fits with 95% CI. Dashed lines indicate the IC5 and IC50 of the dark control condition. Red arrows indicate Oct4 induction upon pulsed illumination (broad range of YAP levels) or chronic light (small range of YAP levels). **B**) Quantification of Oct4 protein levels upon chronic or pulsed (240 min ON/ 60min OFF) light as shown in Fig. 3C. The quantification includes all cells expressing YAP levels >=IC5 (see grey dashed line in panel A). Note that the quantification of Oct4 levels in higher YAP expressing cells (>=IC50) shows higher potency of oscillatory than chronic YAP inputs (see Fig. 3C). Shown are mean +/- SEM, N=7 independent experiments. P values from unpaired Student's t test. **C**) Relation of our YAP-dependent Oct4 phenotypes to the range of endogenously-observed Oct4 levels. Following pluripotency exit, mESCs decrease endogenous Oct4 levels by ~73% over a time course of 4 days post spontaneous differentiation. Our steady-state measurements of Oct4 repression (Fig. 2C) compares to ~83% (low YAP levels) and 4% (high YAP levels) of the Oct4 protein found in naive cells (indicated by horizontal dashed line, red shading represents the SEM of that measurement, N=3 independent experiments). The magnitude of our optogenetic Oct4 induction through oscillatory YAP dynamics (Fig. 3C, pulsed light, 240min ON/ 60min OFF, ΔOct4 = 0.25+/-0.1) is comparable to the amount of Oct4 protein lost within 1d during spontaneous differentiation. Oct4 levels were quantified from IF stainings. Shown are mean +/- SEM, N= 3 independent experiments.

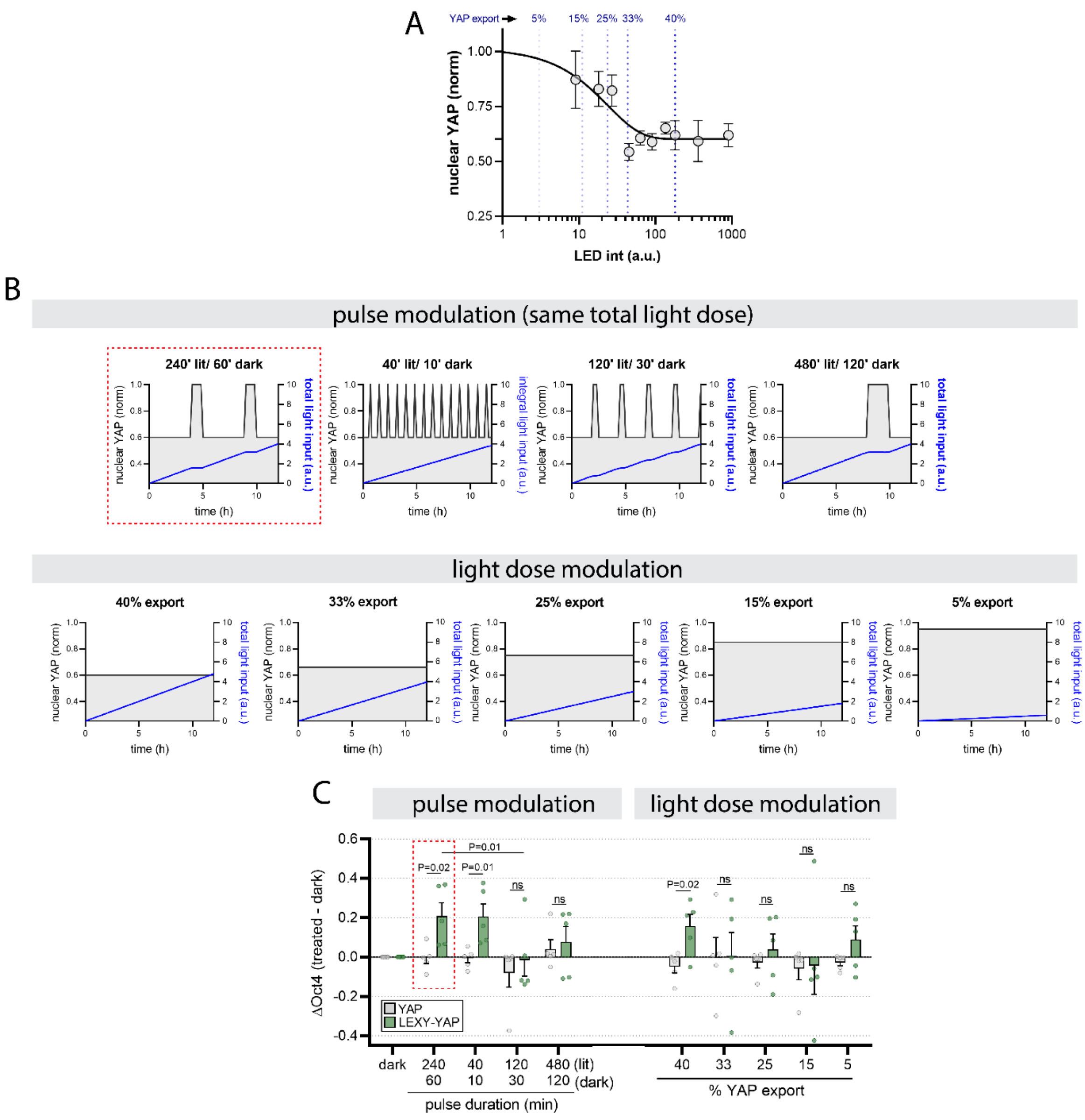


**Supplementary Figure 7 Probing the dynamic decoding capacity of Oct4 by light pulse and dose modulation.**

**A**) Light dose titration of our LEXY-YAP tool by quantification of YAP levels upon illumination with different light intensities. YAP was quantified from IF stainings. Shown are mean +/- SEM, N=5 independent experiments. 40% export refers to the light used throughout our work to achieve full export. Note that cell fixation slightly affects nuclear YAP levels (full light = 40% YAP export) as compared to live cell measurements (Fig. 1I, full light ~ 55% export). **B**) Light profiles used to test the dynamic decoding capacity of Oct4. Shown are illumination profiles for pulse modulation (top row) and light dose modulation (bottom row). Pulse modulation conditions use the same total light dose (right y-axis, blue line) but differ in their pulse durations. Light dose modulation conditions use the same light pattern (chronic light) but differ in the amount of light. 33% chronic YAP export relates to the same total amount of light as the oscillatory 240 min ON/ 60mn OFF light pattern. Light intensities refer to the YAP export values indicated by vertical dashed lines in (A). **C**) Quantification of Oct4 protein induction upon illumination with light pulse and dose modulation patterns shown in (B). Results demonstrate that Oct4 induction by our oscillatory LEXY-YAP pattern (240 min ON, 60min OFF) cannot be explained by decoding of the integral light intensity. Shown are mean +/- SEM, N=5 independent experiments. p values comparing LEXY-YAP vs YAP from two-sided unpaired Student's t test; p values comparing light conditions within the LEXY-YAP group from two-sided paired parametric t test.

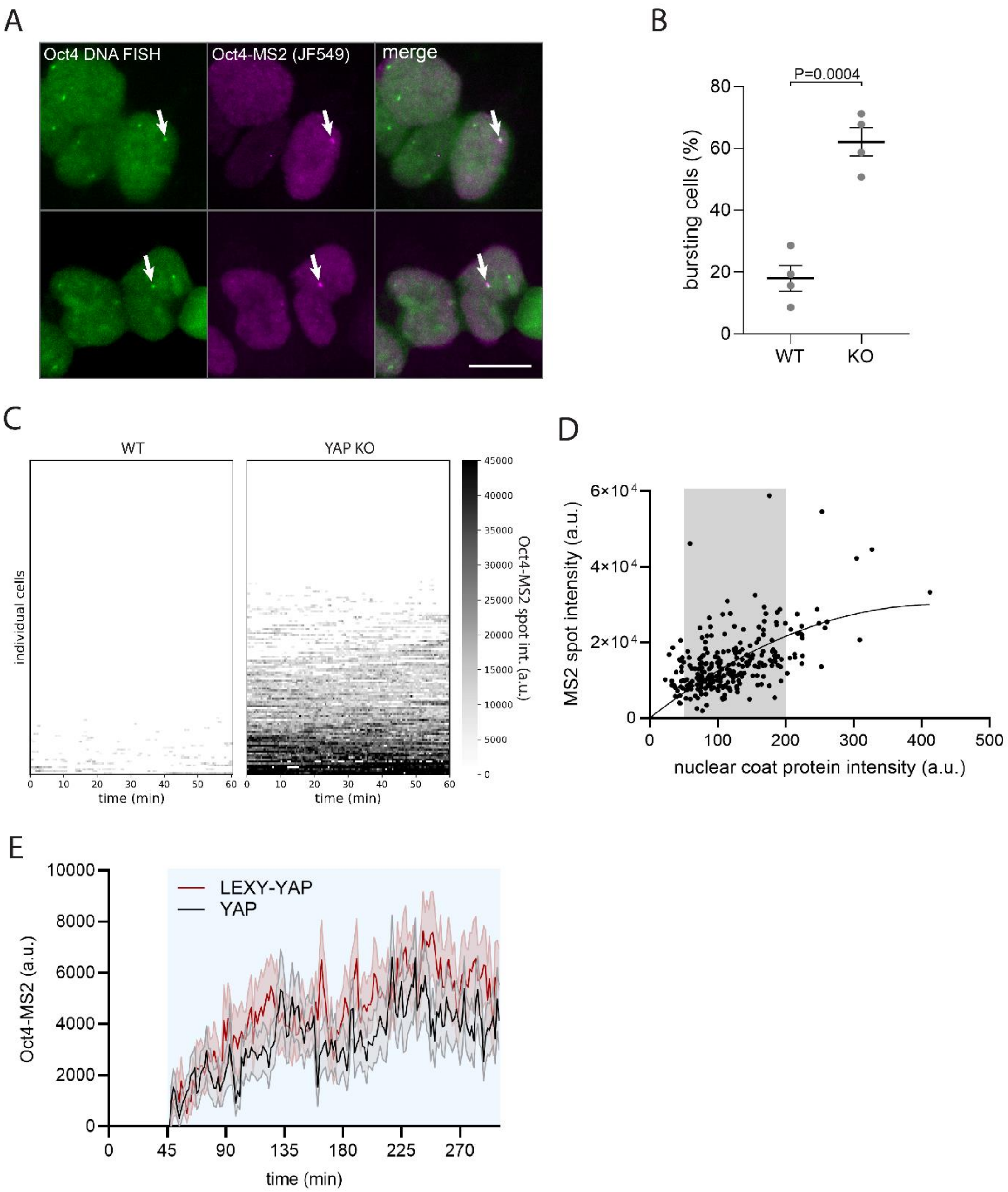


## Supplementary Figure 8 Quantification of Oct4 transcription in YAP KO cells

**A**) Co-localization of Oct4-MS2 spots to the Oct4 locus visualized by Oct4 DNA FISH. White arrows indicate the Oct4 locus and Oct4-MS2 signal. Scale bar: 10 µm. Images are from N=1 experiment. **B**) Quantification of the percentage of Oct4-MS2 cells bursting in WT and YAP KO mESCs at 2d post pluripotency exit. Shown are mean +/- SEM, N=4 independent experiments. **C**) Single cell traces (y-axis) of the Oct4-MS2 signal in WT and YAP KO cells from N=4 independent experiments. **D**) Quantification and polynomial fit of the Oct4-MS2 spot intensity as a function of nuclear coat protein. Only nuclei with coat protein expression range 50-200 a.u. (grey shading) were used for quantifications shown in Fig. 4C, D. The MS2 signal was corrected for coat protein expression differences using the fit. Shown are pooled data from N=6 independent experiments. **E**) Oct4-MS2 transcriptional activity upon light gated nuclear export (blue shading) of LEXY-YAP for cells that are transcriptionally inactive in the dark phase. Shown are mean +/- SEM, N=12 independent experiments.

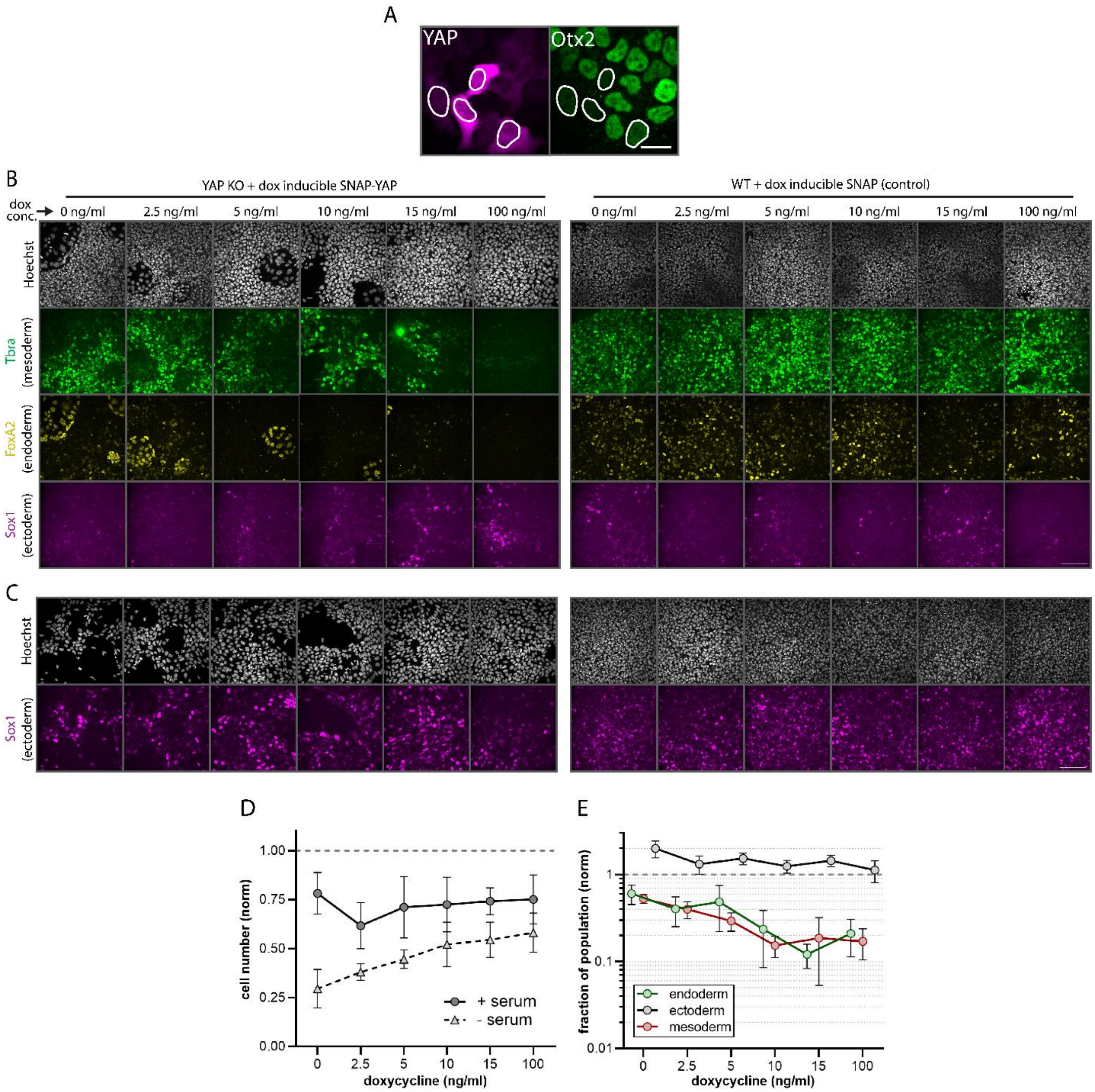


## Supplementary Figure 9 Control of cellular differentiation and proliferation by steady-state YAP levels

**A**) YAP represses cellular differentiation. Shown is an IF co-staining of the early differentiation marker Otx2 and YAP. Nuclei of high YAP expressing cells are outlined in white. Scale bar 20um. **B,C**) IF images showing germ layer markers upon induction of YAP with increasing concentrations of doxycycline as indicated. Cells were grown in spontaneous differentiation media favoring the mesendodermal (B, FBS-based media) or ectodermal (C, N2B27 media) fates. WT mESCs transfected with a doxycycline-inducible SNAP construct serve as control. Scale bar 100µm. Representative images were chosen from N=4 independent experiments. **D**) Comparison of the cell number of mESCs differentiated in FBS containing differentiation media or N2B27 (no serum) shows that YAP induced proliferation is absent in serum-containing media. The data for the serum condition is reproduced from Fig. 6B. E) Validation of the functionality of the LEXY-YAP construct. Quantification of lineage markers from IF images upon induction of LEXY-YAP demonstrate repression of mesendodermal lineage as seen for YAP expressing cells in Fig. 6A. Shown are mean+/- SEM, N=4 independent experiments (D,E). See legend of Fig. 6A for details.

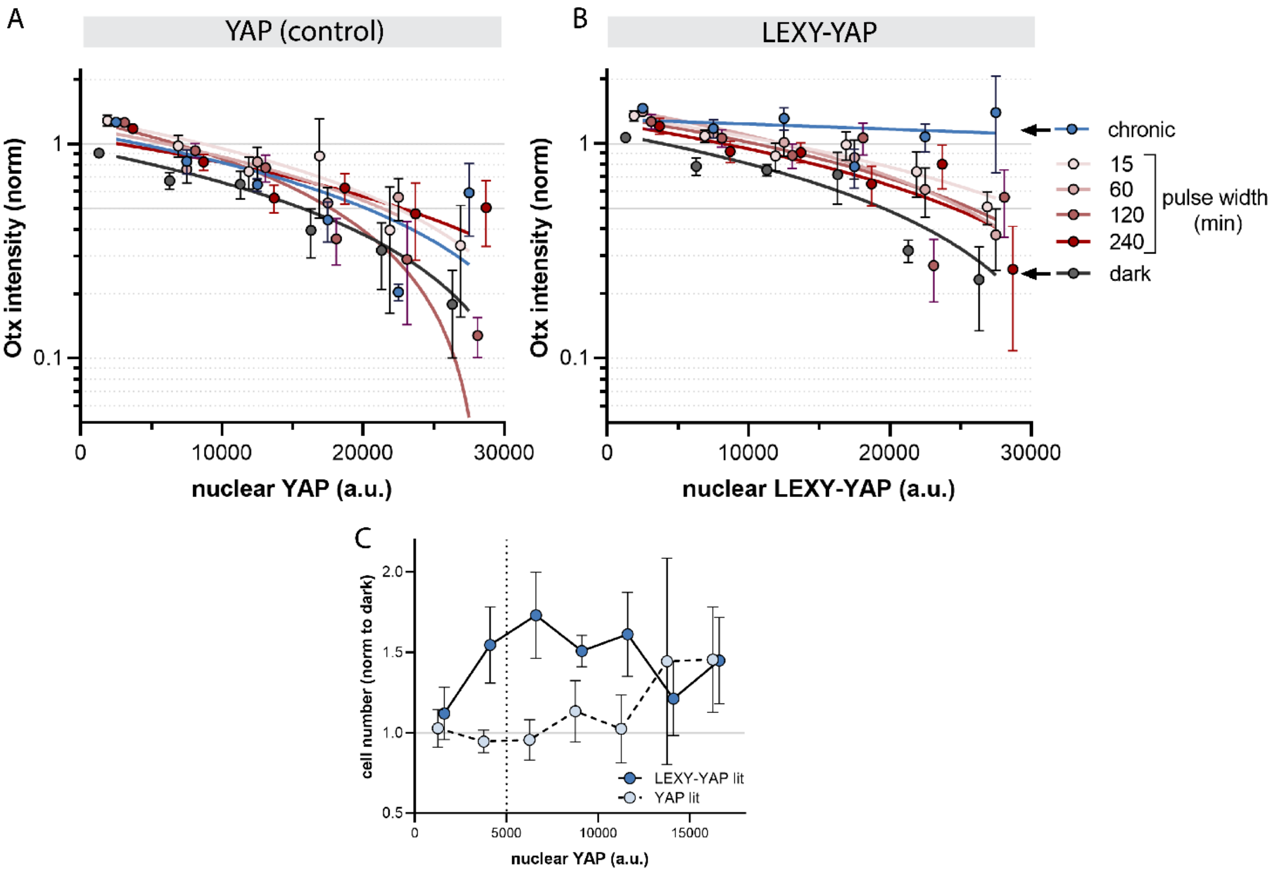


## Supplementary Figure 10 Cellular differentiation requires persistently low YAP levels

**A,B**) Quantification of nuclear Otx2 intensities from IF images as a function of YAP (A) and LEXY-YAP (B) levels. Light illumination with different light patterns demonstrates that persistently low YAP levels (chronic light, blue curve) are required for cellular differentiation as compared to oscillatory YAP dynamics (red curves). For oscillatory light patterns, cells were illuminated with constant YAP export durations of 4h and varying YAP import durations as indicated (legend in B). Data for the chronic and dark condition in (B) are reproduced from Fig. 6C. Shown are mean +/- SEM, N=8 independent experiments. **C**) Quantification of cell numbers from IF images as a function of nuclear YAP levels shows increased cell numbers for intermediate YAP levels (5000-12000 a.u.) upon light illumination. Shown are data for illumination with pulsed (4h OFF, 1h ON) light conditions, see Fig. 6D for comparison to other light patterns. Shown are mean +/- SEM, N=8 independent experiments.

## Supplementary References


1. Kent, W.J., Sugnet, C.W., Furey, T.S., Roskin, K.M., Pringle, T.H., Zahler, A.M., and Haussler, A.D. (2002). The human genome browser at UCSC. Genome Res. *12*, 996–1006.

2. Yeom, Y.I., Fuhrmann, G., Ovitt, C.E., Brehm, A., Ohbo, K., Gross, M., Hübner, K., and Schöler, H.R. (1996). Germline regulatory element of Oct-4 specific for the totipotent cycle of embryonal cells. Development *122*, 881–894.

3. Sandelin, A., Alkema, W., Engström, P., Wasserman, W.W., and Lenhard, B. (2004). JASPAR: an open-access database for eukaryotic transcription factor binding profiles. Nucleic Acids Res. *32*, D91-4.

4. Lian, I., Kim, J., Okazawa, H., Zhao, J., Zhao, B., Yu, J., Chinnaiyan, A., Israel, M.A., Goldstein, L.S.B., Abujarour, R., et al. (2010). The role of YAP transcription coactivator in regulating stem cell self-renewal and differentiation. Genes Dev. *24*, 1106–1118.
