# Supplementary material for: Optogenetic control of YAP reveals a dynamic communication code for stem cell fate and proliferation
Source: Nat Commun. 2023 Oct 30;14:6929. doi: 10.1038/s41467-023-42643-2 (PMC10616176; doi:10.1038/s41467-023-42643-2)
Supplement: Supplementary file 3 — Description of Additional Supplementary Files [file 41467_2023_42643_MOESM3_ESM.pdf]

Description of Additional Supplementary files

Supplementary Movie 1: Endogenous YAP dynamics during mesoderm induction (related to Fig. 1C)

Time-lapse confocal images of endogenous YAP (SNAP-YAP) and quantification of mean nuclear YAP intensity of the outlined nucleus (magenta) show pulsatile nuclear YAP dynamics. Cells were imaged at ~ 1.5d post directed mesoderm induction. Scale bar 10µm.

Supplementary Movie 2: Light-gated control of YAP dynamics (related to Fig. 1H, I)

Time-lapse confocal images of LEXY-YAP expressing mESCs with indicated light illumination shows reversible light-gated YAP export. Blue rectangle indicates illumination phases. Scale bar 20µm.

Supplementary Movie 3: Live imaging of Oct4 transcription in YAP KO cells (related to Fig. 5B and Supplementary Fig. 8B,C)

Maximum projection of time-lapse confocal z-stacks of the Oct4-MS2 transcriptional reporter in WT and YAP KO mESCs shows upregulated Oct4 expression in YAP-depleted cells. Transcription spots are indicated by red circles. Scale bar 10µm.

Supplementary Movie 4: Light-gated control of Oct4 transcription (related to Fig. 4D)

Left: Maximum projection of time-lapse confocal z-stacks of the Oct4-MS2 transcriptional reporter in LEXY-YAP expressing cells shows an adaptive transcriptional response following illumination-induced YAP export. The white arrow indicates the transcription site. The illumination phase is indicated by the blue rectangle. Right: Quantification of the MS2 spot shown in the movie. The vertical line in the graph indicates the onset of the illumination phase. Scale bar 10µm.
