# Supplementary material for: Optogenetic control of YAP reveals a dynamic communication code for stem cell fate and proliferation
Source: Nat Commun. 2023 Oct 30;14:6929. doi: 10.1038/s41467-023-42643-2 (PMC10616176; doi:10.1038/s41467-023-42643-2)
Supplement: Supplementary file 8 — Reporting Summary [file 41467_2023_42643_MOESM8_ESM.pdf]

nature portfolio

Corresponding author(s): Orion Weiner

Last updated by author(s): Oct 11, 2023

Reporting Summary

Nature Portfolio wishes to improve the reproducibility of the work that we publish. This form provides structure for consistency and transparency in reporting. For further information on Nature Portfolio policies, see our Editorial Policies and the Editorial Policy Checklist.

Statistics

For all statistical analyses, confirm that the following items are present in the figure legend, table legend, main text, or Methods section.

n/a	Confirmed	
☐	☒	The exact sample size (*n*) for each experimental group/condition, given as a discrete number and unit of measurement
☐	☒	A statement on whether measurements were taken from distinct samples or whether the same sample was measured repeatedly
☐	☒	The statistical test(s) used AND whether they are one- or two-sided<br>*Only common tests should be described solely by name; describe more complex techniques in the Methods section.*
☒	☐	A description of all covariates tested
☐	☒	A description of any assumptions or corrections, such as tests of normality and adjustment for multiple comparisons
☐	☒	A full description of the statistical parameters including central tendency (e.g. means) or other basic estimates (e.g. regression coefficient) AND variation (e.g. standard deviation) or associated estimates of uncertainty (e.g. confidence intervals)
☒	☐	For null hypothesis testing, the test statistic (e.g. *F*, *t*, *r*) with confidence intervals, effect sizes, degrees of freedom and *P* value noted<br>*Give P values as exact values whenever suitable.*
☒	☐	For Bayesian analysis, information on the choice of priors and Markov chain Monte Carlo settings
☒	☐	For hierarchical and complex designs, identification of the appropriate level for tests and full reporting of outcomes
☒	☐	Estimates of effect sizes (e.g. Cohen's *d*, Pearson's *r*), indicating how they were calculated

Our web collection on statistics for biologists contains articles on many of the points above.

Software and code

Policy information about availability of computer code

Data collection	Micro-Manager version 2.0 gamma, Arduino IDE 1.8.13
Data analysis	Matlab 2020a, Python 3.8.5, Fiji(imageJ) 2.14.0, GraphPad Prism 9.1.0, Nikon NIS-Elements AR.21.01

For manuscripts utilizing custom algorithms or software that are central to the research but not yet described in published literature, software must be made available to editors and reviewers. We strongly encourage code deposition in a community repository (e.g. GitHub). See the Nature Portfolio guidelines for submitting code & software for further information.

Data

Policy information about availability of data

All manuscripts must include a data availability statement. This statement should provide the following information, where applicable:
- Accession codes, unique identifiers, or web links for publicly available datasets
- A description of any restrictions on data availability
- For clinical datasets or third party data, please ensure that the statement adheres to our policy

All data of this study is available in the Source Data file.

Research involving human participants, their data, or biological material

Policy information about studies with human participants or human data. See also policy information about sex, gender (identity/presentation), and sexual orientation and race, ethnicity and racism.

Reporting on sex and gender	n/a
Reporting on race, ethnicity, or other socially relevant groupings	n/a
Population characteristics	n/a
Recruitment	n/a
Ethics oversight	n/a

Note that full information on the approval of the study protocol must also be provided in the manuscript.

Field-specific reporting

Please select the one below that is the best fit for your research. If you are not sure, read the appropriate sections before making your selection.

☒ Life sciences ☐ Behavioural & social sciences ☐ Ecological, evolutionary & environmental sciences

For a reference copy of the document with all sections, see nature.com/documents/nr-reporting-summary-flat.pdf

Life sciences study design

All studies must disclose on these points even when the disclosure is negative.

Sample size	Sample size was not pre-determined. Information on sample size can be found in the Figure legends.
Data exclusions	None unless stated in the Methods section.
Replication	For each experiment, the number of independent experiments (N) is described in the figure legend.
Randomization	Experiments or analysis was not randomized.
Blinding	Data acquisition and analysis was not blinded.

Reporting for specific materials, systems and methods

We require information from authors about some types of materials, experimental systems and methods used in many studies. Here, indicate whether each material, system or method listed is relevant to your study. If you are not sure if a list item applies to your research, read the appropriate section before selecting a response.

Materials & experimental systems

n/a	Involved in the study	
☐	☒	Antibodies
☐	☒	Eukaryotic cell lines
☒	☐	Palaeontology and archaeology
☒	☐	Animals and other organisms
☒	☐	Clinical data
☒	☐	Dual use research of concern
☒	☐	Plants

Methods

n/a	Involved in the study	
☒	☐	ChIP-seq
☒	☐	Flow cytometry
☒	☐	MRI-based neuroimaging

Antibodies

Antibodies used	Sox1 (Cell Signaling Technology, 4194S, 1:200), Tbra (RD Systems, AF2085, 1:200), FoxA2 (Santa Cruz, sc374376, 1:200), Oct4 (Santa Cruz, sc-5279, 1:200), Nanog (Cell Signaling Technology, 8822S, 1:200), YAP (Cell Signaling Technology, 14074, 1:200), Otx2 (R&D Systems, AF1979, 1:200)

Validation	The YAP antibody was validated using the YAP KO line. The Sox1 and Tbra antibodies were confirmed through directed differentiation assay.

Eukaryotic cell lines

Policy information about cell lines and Sex and Gender in Research

Cell line source(s)	E14 mESCs. All genetic perturbations and reporter lines were generated in this study.
Authentication	None of the cell lines were authenticated.
Mycoplasma contamination	Cell lines are regularly tested for mycoplasma and were negative.
Commonly misidentified lines (See ICLAC register)	E14 mESCs are not listed as a commonly misidentified cell line by the ICLAC register.
